# New Polynomial-Based Molecular Descriptors with Low Degeneracy

**DOI:** 10.1371/journal.pone.0011393

**Published:** 2010-07-30

**Authors:** Matthias Dehmer, Laurin A. J. Mueller, Armin Graber

**Affiliations:** Institute for Bioinformatics and Translational Research, Private Universität für Medizinische Informatik und Technik (UMIT), Hall in Tirol, Austria; University of East Piedmont, Italy

## Abstract

In this paper, we introduce a novel graph polynomial called the ‘information polynomial’ of a graph. This graph polynomial can be derived by using a probability distribution of the vertex set. By using the zeros of the obtained polynomial, we additionally define some novel spectral descriptors. Compared with those based on computing the ordinary characteristic polynomial of a graph, we perform a numerical study using real chemical databases. We obtain that the novel descriptors do have a high discrimination power.

## Introduction

The study of specific structural properties of graphs by using algebraic polynomial representations has been a well-known and fruitful concept for several decades [1]–[6]. In particular, graph polynomials have been either used for describing combinatorial graph invariants or to characterize chemical structures by using the coefficients or the zeros of a graph polynomial [4], [7], [8]. As outlined by Gutman [4] and Ivanciuc et al. [9], various topics in mathematical chemistry like Hückel-molecular orbital theory, the theory of aromaticity, and the development of topological indices (e.g., Hosoya index etc.) rely on graph polynomials. Indeed, a very important graph polynomial is the characteristic polynomial of a graph which has been intensely studied by Cvetkovic [10] when exploring structural properties of a graph related to its eigenvalues. Several methods to compute the characteristic polynomial explicitly were also developed [9]. Afterwards, various other graph polynomials [8], [9], [11] such as the Laplacian polynomial, Matching polynomial, Mühlheim polynomial, Distance polynomial and the Wiener Polynomial etc. have been developed for investigating multifaceted aspects of chemical structures.

In this paper, we use the zeros of a novel graph polynomial to derive molecular descriptors. Before outlining the contribution of our paper, we briefly sketch some approaches surveyed by Randić et al. [12] who gave a thorough overview on existing eigenvalue-based descriptors and various applications thereof. An early starting point in this area was initiated by Lovász and Pelikán [13]: They employed the leading positive eigenvalue of the characteristic polynomial as a measure for detecting branching of trees. More advanced concepts to quantify branching of chemical graphs and DNA structures relying on graph-eigenvalues can be also found in Randić's survey [12]. Particularly, the largest and second largest eigenvalues of other graph-theoretical matrices [12], the sum of their positive eigenvalues, the multiplicity of the zero eigenvalue and other spectral indices were also studied [12], [14] to check their ability for serving as molecular descriptors. Importantly, numerical results were reported [12] when applying such descriptors to explore correlations of several physico-chemical properties of chemicals. As a final remark, other eigenvalue-based measures and graph polynomials have been used in various scientific disciplines as well. For instance, graph spectra were employed to derive indices for measuring the structural similarity of networks [15]. In social network analysis and biology, such indices turned out to be useful for defining centrality measures [16], [17]. Estrada [18] derived an eigenvalue-based measure (called the Estrada index) for examining the degree of folding of proteins. More generally, polynomial-based approaches have also been employed to study structural aspects of networks, e.g., see [19], [20].

**Figure 1:**
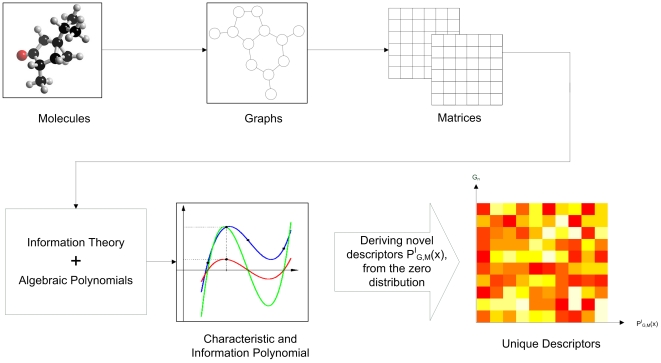
An illustration of the main contribution of this study.

The main contribution of our paper is as follows: Firstly, we define a novel graph polynomial which we call the *information polynomial* of a graph 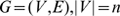. In our sense, we infer this polynomial by using a probability distribution of length 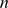 for deriving a graph-theoretical matrix 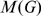 (see Equation (8) in the section ‘Methods’). Secondly, we use the zeros of this polynomial to derive some novel molecular descriptors. Then, we investigate their uniqueness and compare our results with other descriptors. An illustration of our workflow is shown in Figure 1.

## Methods

### The Information Polynomial of a Graph

In this section, we define a novel graph polynomial as well as some spectral molecular descriptors by combining information-theoretic and algebraic methods [21], [22]. To provide measures (descriptors) for quantifying structural information of a graph meaningfully, we develop the notation of the ‘information polynomial’ of a graph. Then, the main idea is to derive a polynomial representation whose coefficients or other characteristics, e.g., its zeros capture structural information of the underlying graph. We want to remark that the expression ‘information polynomial’ has already been used [23], [24] in another context by determining the relative information 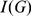 defined as the combinatorial entropy of an oriented graph 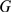.

In particular, it was shown [24] that 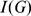 can be expressed by
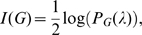
(1)and
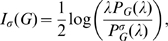
(2)where
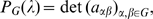
(3)and
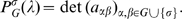
(4)The kind of information polynomial we want to introduce here is notably different from the just mentioned one. The reason why we keep this notation is that the underlying probability distribution as well as the resulting polynomial captures structural information of a graph. In our case, we use an information functional [25]–[27] leading to vertex probability values that can be used to define a graph-theoretical matrix.

We now start with a probability distribution
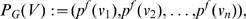
(5)associated to a graph that has already been used to obtain information measures for determining the structural information content of a graph [25]–[28]. The general procedure is as follows: One assigns a probability value to each vertex of a given graph by using a certain information functional 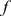 [25]–[27] where 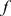 represents a function that maps vertices, or more generally graph elements (when using other invariants), to the non-negative reals. Then, by using Shannon's entropy [29], the structural information content will be defined and interpreted as the entropy of the underlying graph topology [26], [27].

Let's introduce this framework formally. The quantities serving as vertex probabilities are defined by [26]

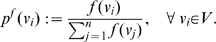
(6)Further, a family of graph entropy measures could be obtained by [26]

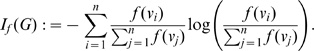
(7)Starting from a graph 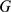 and the probability distribution (see Equation (5)), we now define a matrix for computing an algebraic polynomial which we also call the information polynomial of 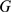. In contrast to the briefly sketched polynomial 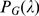 [23], [24], our main goal is to investigate the information polynomial (see Definition (2)) for deriving molecular descriptors from the underlying zero distribution. Further, we remark that our polynomial (see Equation (2)) does not serve as an input for determining a kind of information value. Instead, the structural information of a graph will be captured by the polynomial.

#### Definition 1

*Let* 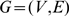 *be a graph and let* 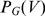 *be the probability distribution assigned to vertex set of* 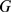. *We define the matrix*

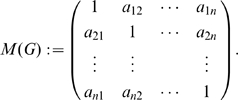
(8)


We set 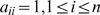 *and*

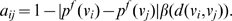
(9)


#### Definition 2

*We define* 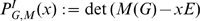. 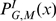 *is called the information polynomial of* 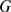.

**Figure 2:**
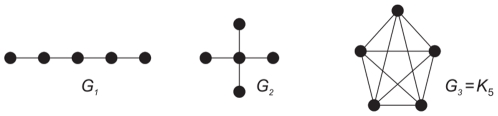
Undirected example graphs 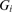.

We remark that starting from Equation (8), (9), we see immediately that 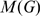 is symmetric, i.e., 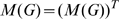. Then, it is clear that all eigenvalues of 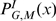 are real [30]. Also, we point out that to comprise the connection between 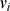 and 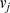, we use a function 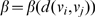 (see Equation (9)) depending on the shortest distance between the corresponding vertices. To calculate 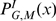 for some simple graphs exemplarily, we consider Figure 2 and set
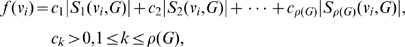
(10)and 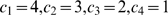 and 
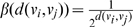
 for determining the probability distribution 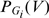. 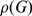 is the diameter of 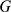. We yield
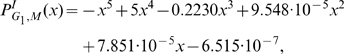
(11)

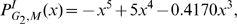
(12)

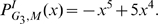
(13)and the corresponding sets of zeros are
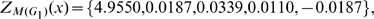
(14)

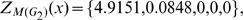
(15)

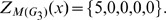
(16)In particular, we always get

#### Proposition 1



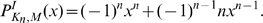
(17)
*The positive eigenvalue is* 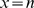.

### Novel Descriptors

In the following, we define some novel descriptors derived from the just introduced polynomial. The idea is to use the underlying zero distribution of 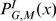.

#### Definition 3

*Let* 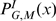 *be the information polynomial of* 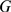 *and let* 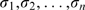 *be its real zeros.*

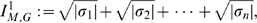
(18)

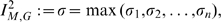
(19)

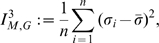
(20)

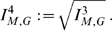
(21)


## Results and Discussion

The aim of this section is to evaluate the just defined descriptors (see previous section) in terms of their uniqueness (degeneracy) [31], [32]. This property of a molecular descriptor relates to the ability to distinguish graphs as uniquely as possible by calculating the underlying graph measure. In general, a descriptor is called degenerated if there are at least two non-isomorphic graphs possessing the same value. For instance, concrete studies [32], [33] to explore this problem by using isomeric and lattice structures were performed. Particularly, Bonchev et al. [7] investigated the degeneracy of information-theoretic indices relying on Shannon's entropy. Further, Todeschini et al. [34] evaluated several known non-information-theoretic and information-theoretic topological indices based on a large set of real chemical compounds. Another important problem is to quantify the degree of degeneracy of a given index. For this, a sensitivity measure as well as an information-theoretic measure have been developed by Konstantinova [32], [33] and Todeschini et al. [34], respectively.

### Datasets

- AG 3982: To create this database, we used the benchmark database called Ames mutagenicity [35], [36]. This database has originally been used for predicting the mutagenicity of chemical compounds [36]. Note that the Ames database has been created from six different public sources [35], [36]. By starting from the original database Ames mutagenicity [35], [36] containing 6512 chemical compounds, we created AG 3982 by filtering out isomorphic graphs based on the software SubMat [37]. Finally, this procedure resulted in 3982 structurally different skeletons, that is, all atoms and all bonds are considered as equal. It holds 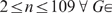 AG 3982.
- MS 2265: To create this dataset, the mass spectral database NIST [38] was used [27]. The entire database contains approximately 100000 chemical structures from organic compounds [27]. A set of 4000 structures with 4 to 19 non-hydrogen atoms (vertices of the graph) have been randomly selected from the database. Finally, 2265 of them have different skeletons (all atoms and all bonds considered to be equal, all hydrogen atoms removed). It holds 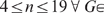 MS 2265.

### Software and Technical Processing of Graphs

We implemented the novel descriptors and performed all involved calculations by using the programming language R [39] and the freely available library ‘graph’. In order to generate the underlying graph structures representing chemical compounds, we used Molfile format [40]. We remark that the graphs originating from AG 3982 were originally available in Smiles format. Thus, we converted them to Molfile format (SDF) using a Python procedure. The graphs from MS 2265 were originally available as Molfiles. In both cases, a R procedure was developed to create the adjacency matrices as well as the matrices 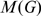 for calculating the novel descriptors from the available Molfiles. To determine their degree of degeneracy (sensitivity measure) in the next section, we use Equation (10) and exponentially decreasing parameters chosen as
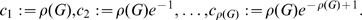
(22)


### Uniqueness of the Descriptors

We now interpret the results we have obtained from calculating the sensitivity index [32]

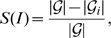
(23)of a descriptor 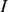 as follows. Here, 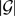 is the cardinality of a given set of graphs 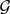 and 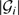 denotes the number of graphs 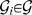 which can not be distinguished by an index 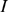. In Table 1, we see the results when using the novel descriptors based on the underlying information polynomial 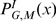. In Table 2, we summarize the results when using the same descriptors (these are 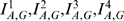) based on the ordinary characteristic polynomial 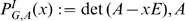 is the adjacency matrix of 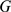. For both polynomials 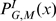 and 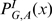, we observe that the sums of the square roots of the moduli of their real zeros (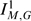 and 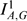) have a high discrimination power. But by evaluating the characteristic polynomial instead of the information polynomial, one can see that the sensitivity values are a little less. Further, 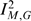, 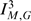 and 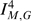 also do have a high discrimination power based on their sensitivity values shown in Table 1. As expected and known [41], the Wiener Index [42] and Randić Index [43] do possess a relatively high degree of degeneracy leading to low sensitivities. However, it is known that, for instance, information-theoretic measures [14], [25] do often have a high discrimination power. For comparing such measures with ours, we also calculated the sensitivities of the two entropic measures
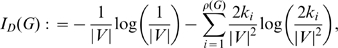
(24)

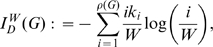
(25)which were developed by Bonchev [25]. Here, 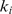 stands for the occurrence of the distance value 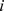 in the distance matrix 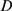. We see in Table 1 that the resulting sensitivities are much better than the ones obtained by calculating 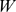 and 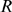.

**Table 1:** Calculation of sensitivity index 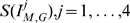 for two chemical databases.

Descriptor 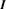	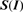 for MS 2265	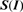 for AG 3982
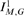	0.999117	0.998995
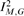	0.997351	0.994224
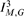	0.998234	0.997489
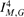	0.988521	0.976645
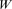	0.067108	0.343046
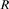	0.232546	0.402562
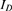	0.859602	0.938724
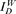	0.883885	0.947513

**Table 2:** Calculation of sensitivity index 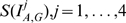 for two chemical databases.

Descriptor 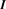	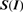 for MS 2265	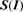 for AG 3982
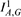	0.928918	0.964591
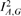	0.699338	0.834003
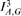	0.000883	0.000883
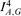	0.01557	0.01557

Now, we evaluate the discrimination power of the polynomial-based descriptors. Corresponding to the fact that the characteristic polynomial of a graph is degenerated [41] (i.e., many non-isomorphic graphs have the same spectra), it is not surprising that the sensitivity values for 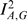, 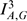 and 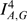 are much less (for both MS 2265 and AG 3982) than in case of considering the information polynomial. In fact, the discrimination power of 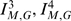 when taking the characteristic polynomial into account (i.e., they become to 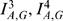) is very little. Finally, we also remark that 
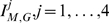
 are less degenerated than 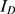 and 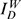.

Also, Table 3 shows some characteristics of the spectra concerning MS 2265 and AG 3982. For instance, 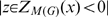 stands for the number of zeros (eigenvalues) of the information polynomial less than zero. 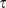 stands for the minimal eigenvalue and 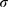 for the maximal one. The table gives information about the distribution of the zeros with respect to the used databases. For both MS 2265 and AG 3982, we get the result that approximately 70% of the eigenvalues are positive, 25% are negative.

**Table 3:** Characteristics of the spectra concerning MS 2265 and AG 3982.

	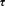	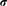	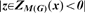	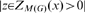	% 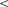 0	% 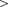 0
MS 2265	−0.02	18.99	7798.00	21419.00	0.25	0.69
AG 3982	−0.02	109.00	20539.00	56159.00	0.25	0.70

As conclusive remarks, the obtained results (in particular, see Table 1) show that the spectrum of the information polynomial seems to contain useful information for defining special molecular descriptors. In particular, we infer that the information polynomials of our chemical graphs are less degenerated than the corresponding characteristic polynomials. From this, we also conclude that some of the novel measures (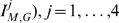) are suitable for characterizing (large) graphs structurally because they encode structural information uniquely.

### Summary and Outlook

In this paper, we introduced a novel graph polynomial and derived some descriptors by using the underlying zero distribution. We summarize the main findings of our paper and some future ideas as follows:

- We started from the idea to use the probability distribution 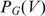 (see Equation (5)) possessing 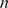 vertex probabilities which depend on an information functional [26] 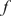. Instead of using 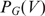 to derive entropic measures for graphs [26], [27], this probability distribution served as starting point to derive a graph-theoretical matrix finally leading to the definition of the information polynomial.
- So far, spectra of graphs have already been used to characterize chemical graphs [41], [44]. However, it is known that graph spectra (with respect to the ordinary characteristic polynomial) are degenerated, that means, many non-isomorphic graphs do have the same characteristic polynomials and spectra. For this reason, we investigated the ability of the information polynomial and the derived descriptors to discriminate graphs structurally. In particular, we found that the descriptors based on the information polynomial do have a high discrimination power by evaluating the sensitivity index due to Konstantinova [32]. But we emphasize that the achieved results depend on the used datasets (see Section ‘Datasets’). As future work, we want to further develop spectral descriptors and evaluate them by using large sets of chemical and bio-chemical structures.
- It is well-known that various topological descriptors have been successfully used for structure-oriented drug design [45]–[47]. Typically, the descriptors are used to predict a biological or physico-chemical property by taking the structure of the underlying molecule into account. The next step in this direction would be to apply our novel descriptors to further datasets for evaluating their usefulness for solving QSAR/QSPR problems. As a strong point, we have proven that our novel indices encode structural information uniquely that is generally a desirable property of a molecular descriptor. This could indicate their usefulness when applying the measures in drug design, e.g., for classifying chemical structures. However, we note that also degenerated measures were found to be successful for QSAR modeling, see [48].
- There is a considerable body of literature dealing with examining the zeros of graph polynomials [49]–[51]. For example, Woodall [51] examined the zeros of chromatic and flow polynomials and determined zero-free regions thereof. When giving [50] a survey on counting hypercubes, Kovše [50] also sketched some results concerning the zeros of cube polynomials. Jackson [49] surveyed results and conjectures dealing with the zero distribution of chromatic and flow polynomials of graphs, and characteristic polynomials of matroids. Note that a recent overview on such results was given by Ellis-Monaghan et al. [6]. As future work, we want to study similar questions concerning the information polynomial. This would involve examining problems such as:
  - Deriving special estimations for the largest positive zero of 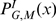.
  - Deriving special bounds (leading to intervals containing the real zeros of 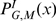) for the moduli of the real zeros of 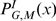.
  - Exploring the location of the zeros for similar types of graph polynomials. Particularly, we want to derive special bounds for special graph classes.
- As mentioned, the largest positive eigenvalue of trees has been used as a measures for branching [13] where Bonchev [52] gave an overview on the concept of branching and measures to quantify it [52]. In the future, investigate the largest positive eigenvalue of our information polynomial in depth and compare the results with other graph polynomials.
